# A web-based interactive tool to improve breast cancer patient centredness

**DOI:** 10.3332/ecancer.2016.659

**Published:** 2016-07-26

**Authors:** Alessandra Gorini, Ketti Mazzocco, Haridimos Kondylakis, Gordon McVie, Gabriella Pravettoni

**Affiliations:** 1 Department of Oncology and Haemato-Oncology, University of Milan, Via Festa del Perdono 7, 20100 Milan, Italy; 2 European Institute of Oncology, Via G. Ripamonti 435, 20100 Milan, Italy; 3 Computational BioMedicine Laboratory, Institute of Computer Science, Foundation for Research and Technology - Hellas, N. Plastira 100, Vassilika Vouton, GR-700 13 Heraklion, Crete, GR-700 13, Greece

**Keywords:** patient empowerment, patient–physician communication, breast cancer, personalised medicine, web-based tool

## Abstract

The uniqueness of a patient as determined by the integration of clinical data and psychological aspects should be the aspired aim of a personalized medicine approach. Nevertheless, given the time constraints usually imposed by the clinical setting, it is not easy for physicians to collect information about the patient’s unique mental dimensions and needs related to her illness. Such information may be useful in tailoring patient–physician communication, improving the patient’s understanding of provided information, her involvement in the treatment process, and in general her empowerment during and after the therapeutic journey. The primary objective of this study is to evaluate the effect of an interactive empowerment tool (IEm) on enhancing the breast cancer patient–physician experience, in terms of increasing empowerment, i.e. by providing physicians with a personalised patient’s profile, accompanied by specific recommendations to advise them how to interact with each individual patient on the basis of her personal profile.

The study will be implemented as a two-arm randomised controlled trial with 100 adult breast cancer patients who fill in the ALGA-BC questionnaire, a computerised validated instrument to evaluate the patient’s physical and psychological characteristics following a breast cancer diagnosis. The IEm tool will collect and analyse the patient’s answers in real time and send them, together with specific recommendations to the physician’s computer immediately before physician’s first encounter with the patient. Patients will be randomised to either the intervention group using the IEm tool or to a control group who will only fill in the questionnaire without taking advantage of the tool (physicians will not receive the patient’s profile).

The proposed approach is supposed to improve the patient–physician communication leading to increased patient participation in the therapeutic process as a consequence leading to improvement in patient empowerment and personalisation of care.

## Background

In recent years there have been significant moves towards attempting to transform the traditional paternalistic approach, in which the physician is parental, recommending what he or she feels is best for the patient, to an informative patient-centred approach. Such an approach considers it to be essential for the patient to be actively involved and to participate in the decision-making processes concerning treatment in order to assure that decisions are consistent with his/her values, preferences, and needs. Nowadays, promoting a patient-centric approach and improving patient empowerment is considered not only a matter of health quality improvement, but also an essential component of healthcare delivery systems [1].

Available data indicate that there are multiple ways of empowering patients ranging from health literacy and awareness campaigns to a more sophisticated approach that of providing tools that enable them to self-manage or monitor their health conditions [2–5]. Among these possible options, we argue that a promising approach consists of promoting personalised interventions aimed at providing a more effective and efficient communication channel between the healthcare providers and the patients. In order to make this possible, providers should be prepared to inform and support their patients armed with an understanding of their knowledge gaps, personal values about possible outcomes, and treatment preferences [6, 7]. However, clinical consultations usually take place under conditions of limited time and resources where physician’s talk overwhelms patient’s status, preferences, and considerations [8]. As a consequence, in a number of instances, what the healthcare provider assumes is different from what actually a patient understands or needs. This can cause a lack of compliance to treatment instructions indicating that informed health management requires conscious and active participation of the patients at least because they are the master of their own body. On the contrary, different studies have shown that patients who are actively engaged in their own healthcare have better outcomes and lower costs [9, 10]. Enabling patients to be in charge of their own care is one of the main focus of the broad concept of ‘patient empowerment’ that can be realised when patients are encouraged to take an active part in their own health management, become self-determining agents with some control over their own health and care, rather than passive recipients of healthcare services.

A number of factors can facilitate this process, including the use of information and communication technology systems (ICTs) [11]. The impact of ICTs on healthcare is twofold: from one side they can be useful to provide a vast amount of informational resources openly available and to connect patients and professionals who are not physically present in the same place (i.e. internet-based services); from the other side their computational capabilities can be used to analyse, almost in real time, individual patient’s data that might be useful to provide them with appropriate knowledge for making informed decision and/or to provide their physicians with information which is relevant to promote their healthcare in a personalised way. This latter option is crucial to fill the gap between optimal conditions, in which the physician has all the relevant information about the patient (including the non-medical ones, such as those coming directly from patients about how they understand, function or feel in relation to their health condition, and its treatment) and the actual encounters characterised by limited time and information.

Starting from these considerations, we have developed an Interactive Empowerment (IEm) tool aimed at enhancing physician–patient experience by providing physicians a personalised patient’s profile, based on her psychological and cognitive characteristics, accompanied by specific recommendations on how to deal with each patient in the way that best fits with her needs. The IEm was developed in the context of the FP7 project ‘P-Medicine’ [http://p-medicine.eu/], which aspires to create an infrastructure that will facilitate the translation from current medical practice to personalised medicine. The IEm tool is based on the ALGA-BC questionnaire [12], a recent validated instrument specifically developed to perform a brief evaluation of the breast cancer patients’ psychological status. As soon as the patient completes the questionnaire, her answers are automatically elaborated and put together to create a patient’s profile, and it is sent to the physician’s computer. If provided to the physicians at the very beginning of the visit, these informations will be crucial in that it will help them find a tailored way to communicate with the patient. Moreover, in order to help physicians to correctly interpret the patient’s score, short explanations, and recommendations are provided to help them to find the best way to interact with the patient. Since patient empowerment can be reached through improved patient participation in the clinical process, we argue that a more effective and personalised interaction between patient and physician may have a key role in promoting it.

The primary purpose of the present trial is to test the efficacy of a web-based patient profiler tool (the IEm tool) to improve patient empowerment through a better understanding of the individual patient’s characteristics by the physicians. The secondary question is whether a better interaction between patient and physician increases the patients’ understanding and their ability to retain information, and also if it enhances the patient’s and physician’s satisfaction. Finally, we will investigate the physicians’ perceived usefulness, perceived ease of use, and user acceptance of the tool by means of the adapted form of Technology Acceptance Model Questionnaire.

## Methods

A randomised control trial will be implemented with 1) an experimental group in which the patient fills in the questionnaire and the physician receives her profile together with short recommendations on how to tailor the communication and interaction with the patient, and 2) a control group in which the patient fills in the questionnaire, but the physician does not receive either the profile or the recommendations. The reason why the questionnaire is provided to both groups (experimental and control) is to exclude any confounding variables that might affect patient empowerment beyond the personalisation of doctor–patient interaction.

### Participants

Participants will be women with breast cancer aged 18 to 75 years diagnosed with primary breast cancer who undergo a radical surgery. Patients with recurrent breast cancer or overt psychiatric illness that could interfere with the measurement of psychological variables will be excluded from the study. The study will be conducted at the European Institute of Oncology (IEO) in Milan, Italy, and patients will be recruited via medical oncologists operating in the same Institute.

The present trial is part of the European Project ‘P-Medicine’, whose final aim is to create a complex tool to provide a personalised treatment to cancer patients.

### Inclusion/exclusion criteria

The inclusion criteria are the following:

1. The patient is aged 18 to 75 years at randomisation.
2. The patient has been diagnosed as having a primary breast cancer requiring a radical surgery
3. The patient is able to understand the Informed Consent Form, and understand study procedures.
4. The patient has signed the Informed Consent Form.

The exclusion criteria are as follows:

1. The patient has a recurrent breast cancer diagnosis.
2. The patient has an overt psychiatric illness that would interfere with the measurement of the psychological variables included in the questionnaire.
3. The patient is currently participating in another clinical trial relating to the breast cancer treatment.

### Experimental procedures

Patients will be asked to come to the hospital 30 minutes prior to their first visit with the physician, and after obtaining the informed consent she should fill in the ALGA-BC questionnaire using an iPad. The whole process will be supervised by a research assistant who eventually answers questions concerning the questionnaire itself and the use of the electronic device. The iPad will be wirelessly connected to the internal hospital network, so that as soon as the patient finishes completing the questionnaire, the answers will be automatically collated and directly sent to the physician’s computer.

### Tool description

In order to support communication, interaction, and information delivery process from doctor to patient a smart environment has been implemented. This environment consists of two key components: The ALGA-BC questionnaire and the profiler component [13, 14].

### The ALGA-BC questionnaire as a profiling instrument for breast cancer patients

The ALGA-BC questionnaire is a novel multidimensional questionnaire that assesses the breast cancer patient’s physical and mental aspects in order to create a patient’s profile to facilitate communication, interaction, and information delivery process from the doctor to the patient. In particular, ALGA-BC is intended to foster the shared decision making process between patients and physicians and to empower the patient by actively involving him/her in the medical decision-making process. The questionnaire includes 29 questions divided in eight functioning scales labeled: ‘global self-rated health’, ‘perceived physical health’, ‘anxiety’, ‘self-efficacy’, ‘cognitive closure’, ‘memory’, ‘body image’, and ‘sexual life’. Once completed, each patient receives a numerical score for each factor that contributes to generate the final profile.

**Figure 1.**
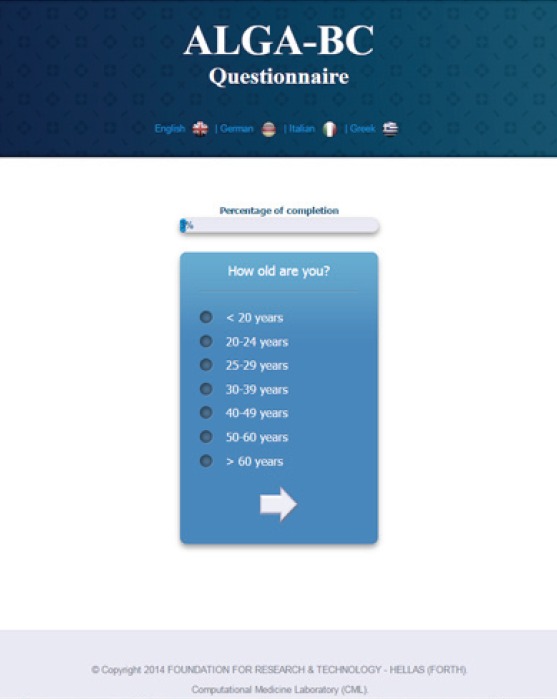
A screenshot from the ALGA-BC questionnaire. The figure shows one of the questions included in the ALGA-BC questionnaire as seen by the patient.

The web-based version of the ALGA-BC [http://thor.ics.forth.gr:8580/QuestionnaireApp/] (Figure 1) is available in four languages (Italian, English, German and Greek), and it has been developed to be compatible with all web browsers and mobile devices.

### The profiler

**Figure 2.**
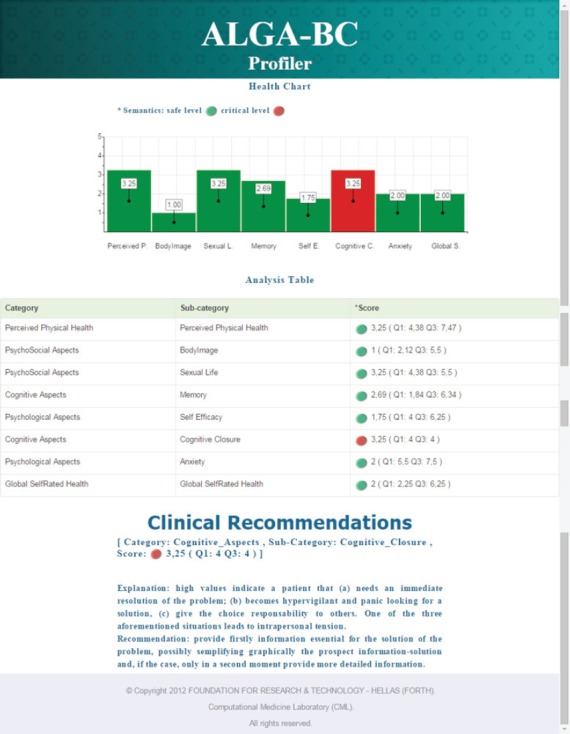
*A screenshot from the profiler application*. Accessing the profiler, the physician can see the ‘Health Chart’, that is a graphic representation of the patient’s scores in the different areas; the ‘Analysis Table’, that indicates the patient’s scores and the range of comparison values (quartiles calculated in the comparison population); and the ‘Clinical Recommendations’, a brief explanation of the factor(s) in which the patient obtained an abnormal score and a practical recommendation to manage it.

The profiler is a web application [http://thor.ics.forth.gr:8580/ProfilingServer/] that analyses the patients’ answers to the ALGA-BC questionnaire and creates their personal profile in real-time. Through the profiler the doctor can easily search for a specific patient and visualise her results. The scores for each factor included in the questionnaire (‘global self-rated health’, ‘perceived physical health’, ‘anxiety’, ‘self-efficacy’, ‘cognitive closure’, ‘memory’, ‘body image’, and ‘sexual life’) are automatically calculated and presented in a graphic mode. Scores that deviate more than two standard deviations from the average population are represented using red columns (or red buttons) (see Figure 2). The average population can be a sample of breast cancer patients or a sample of healthy subjects, depending from the need and the interest of the physician. When one or more of the patient’s scores deviate from average, one or more recommendations are provided to the physician together with a short explanation of the corresponding factor(s).

### The psychological recommendation construction

The cognitive and psychological factors appearing in the profiler tool represent the level of mental resources a person can allocate to information processing. Alteration(s) in one or more of them can affect selection, memorisation, and the consequent understanding and use of relevant data, messages, and prescriptions provided to the patients by the physician.

First, a search of the existing literature was conducted to find relevant studies examining the influence of cognitive and psychological factors measured by the ALGA-BC questionnaire on a person’s information processing [15–21]. Then, brief explanations and recommendations will be created for each factor to help physicians understand what the critical values imply and to guide them in tailoring a more effective communication with the patient.

On the other side, the use of such recommendations is supposed to build up a context in which the patient will be facilitated in focusing on information essential to comprehensively understand her own health condition, the treatment options, and in focusing on aspects that might increase the likelihood of positive outcome.

### Measures

To verify the effect of the intervention, patient empowerment will be measured in the experimental versus the control group using quantitative and qualitative measures. The quantitative measure consists of a multilingual self-reported questionnaire (Questionnaire for Patient Empowerment Measurement; http://www.sustainsproject.eu/sustainsproject/attachment/d32v10questionnaireforpatientempowermentassessment.pdf) originally developed to assess the impact of patients’ access to their Electronic Health Records (EHR) and related services on patient empowerment. The questionnaire consists in 19 items whose answers range from Never (0) to Always (10), and is aimed to evaluate the effect of the electronic patient profile generated by the ALGA tool on three different patient empowerment dimensions, i.e. patient knowledge, patient control, and patient participation.

The qualitative measure of empowerment includes a questionnaire that measures the comprehension level relative to the information provided by the doctor comparing the patients’ knowledge about their own disease before and after the visit with the oncologist. A comparison between information provided by the doctor and recalled by the patient will be performed: two independent researchers will assist the doctor–patient encounter recording provided information and will record the patient’s recall respectively. Finally, satisfaction about the physician–patient interaction will be evaluated using a 10-point Likert scale (1 = no satisfaction at all; 10 = extreme satisfaction) both in physicians and patients, in order to test differences, if any in the perception of the two samples.

We will also investigate the physicians’ perceived usefulness, perceived ease of use, and acceptance of the tool using the adapted form of Technology Acceptance Model Questionnaire.

### Sample size and power calculations, and randomisation

A trial of 50 participants per arm is sufficient to detect a significant difference (alpha = 0.05, two-sided test) in the empowerment measurement between arms with 80% power.

Group randomisation is to be conducted by the European Institute of Oncology. Participants will be blinded to the nature of the intervention they are receiving. While for reasons of evaluating and contacting participants the research assistant cannot be blinded, the primary analyst will be blinded to participant allocation.

### Proposed analytic strategy

The number of participants completing the questionnaire will be tabulated. The number of participants withdrawing during the course of the study will be tabulated with reason for withdrawal where known. The data will be screened to detect outliers and assess the distributions of the primary and secondary outcome variables. Outliers will not be excluded from analyses unless there is independent evidence that their scores are not valid.

Demographic and other clinical patient characteristics (age, sex, education, etc.) recorded on screening will be tabulated too. Descriptive statistics will include n, mean, standard deviation, median, minimum, and maximum. Differences in demographic and other patient characteristics in the experimental and control subjects will be identified.

### Intervention effectiveness

The final measure of empowerment of each patient corresponds to mean score difference obtained in the Patient’s Empowerment Questionnaire and computed between the total score in the nine items before and the nine items after the visit. Correlation between perceived empowerment and effective comprehension will be used as a measure of coherence in empowerment. For the primary outcome of empowerment, assuming normal variable distributions, the intervention arms will be compared to the control arm using t-tests and one-way analysis of covariance using study arm as the between-subjects factor and the ALGA-BC scores as the covariate [22]. Similar analyses will be conducted for the secondary outcome variables and in all cases effect sizes reported. Where variable distributions do not initially permit the use of parametric inference tests, variables will be subjected to mathematical transformation to achieve normality. In cases where transformations are not effective, non-parametric tests on difference scores will be used.

### Ethics

The trial has received a favourable ethical opinion by the hospital internal ethical committee ‘Comitato Etico degli IRCCS Istituto Europeo di Oncologia e Centro Cardiologico Monzino’ (R599 – IEO S645/311).

## Expected results and discussion

This randomised controlled trial is aimed to investigate the effects of an innovative web-based profiling tool on the promotion of patient empowerment. At the same time, we want to analyse if the provision of specific recommendations based on the patient profile makes a physician more comfortable during the first encounter with a new patient and more confident in the possibility to improve the shared decision making process through an improved understanding and participation of patients.

We hypothesise that an increase in the knowledge of the patient from the physician’s side might result in a better and more efficient relationship between the two. Extending their knowledge from physical symptoms and clinical signs to the psychological, cognitive, and emotional aspects of breast cancer patients, physicians are supposed to improve their ability to understand the patients’ needs thereby consequently providing a more tailored and personalised intervention and improving patient empowerment.

Questionnaires that investigate patients’ quality of life and other related psychological aspects are a common custom in clinical practice. However, to the best of our knowledge this is the first attempt to develop a web-based tool that not only automatically creates a personalised profile for each patient, but also help physicians through pre-defined guidelines and recommendations, to interpret results and personalise their intervention according to it.

The challenges for this trial include two main issues. The first one has to do with the potential of the tool to really increase patient empowerment, while the second depends on the physicians’ availability and adherence to the use of such a tool. Even if a substantial amount of work has been put into creating a usable tool with a very friendly-user interface, we expect that some physicians will be resistant to introducing such a tool to their daily clinical activity.

Moreover, since the present study is focused only on breast cancer patients, further parallel work is required to explore the feasibility and effectiveness of such a tool for different kinds of cancer and other pathologies.

## Conclusions

In conclusion, the proposed approach is expected to improve the patient–physician communication leading to increased patient participation in the therapeutic process with a consequent improvement in patient empowerment and personalisation of care.

## Conflicts of interest

The authors declare that they have no competing interests.
